# Epidermiology of adverse drug reactions (ADRs) in the elderly

**DOI:** 10.1186/2045-7022-4-S3-P129

**Published:** 2014-07-18

**Authors:** Kim Ju-Young, Kyung-Hwan Lim, Han-Ki Park, Kang Hye-Ryun, Chang Yoon-Seok

**Affiliations:** 1 Seoul National University Bundang Hospital, Internal medicine, Korea, Republic of; 2 Seoul National University College of Medicine, Internal medicine, Korea, Republic of

## Introduction

Several circumstances contribute to the greater propensity for adverse drug reactions (ADRs) in the elderly, including use of multiple drugs and pharmacokinetic and pharmacodynamic alterations intrinsic to aging. We sought to evaluate the characteristics of ADRs in the elderly compared with younger adults.

## Methods

ADRs were collected from a spontaneous reporting system at Seoul National University Hospital from February 2010 to September 2013. We analyzed causative drugs, clinical manifestations and severity of ADRs.

## Results

A total of 15,541 ADRs was reported in patients greater than or equal to 18 years. Common causative drug category included nervous system, neoplastic, anti-infectives. The prevalence of ADRs due to respiratory drugs and cardiovascular drugs was higher in the elderly group (¡Ã 60 years) than in other groups. The most prevalent clinical types were gastrointestinal disorders and skin and appendage disorders. Psychiatric disorder, cardiovascular disorder, hematologic disorder and genitourinary disorder were more prevalent in the elderly group than in other groups. The proportions of severe ADRs were higher in the elderly groups or in male patients.

## Conclusions

Elderly patients seemed to be more susceptible to ADRs. Further efforts to understand, management and prevent the ADRs in the elderly are required.

